# β-Sitosterol Protects against Carbon Tetrachloride Hepatotoxicity but not Gentamicin Nephrotoxicity in Rats via the Induction of Mitochondrial Glutathione Redox Cycling

**DOI:** 10.3390/molecules191117649

**Published:** 2014-10-30

**Authors:** Hoi-Shan Wong, Ji-Hang Chen, Pou-Kuan Leong, Hoi-Yan Leung, Wing-Man Chan, Kam-Ming Ko

**Affiliations:** Division of Life Science, Hong Kong University of Science and Technology, Hong Kong, China; E-Mails: bchelenwong@ust.hk (H.-S.W.); cjh@ust.hk (J.-H.C.); eriol@ust.hk (P.-K.L.); hoiyan@ust.hk (H.-Y.L.); wingman@ust.hk (W.-M.C.)

**Keywords:** Cistanches Herba, β-sitosterol, carbon tetrachloride hepatotoxicity, gentamicin nephrotoxicity, glutathione redox cycling, mitochondria

## Abstract

Previous findings have demonstrated that β-sitosterol (BSS), an active component of Cistanches Herba, protected against oxidant injury in H9c2 cardiomyocytes and in rat hearts by enhancing mitochondrial glutathione redox cycling, possibly through the intermediacy of mitochondrial reactive oxygen species production. We therefore hypothesized that BSS pretreatment can also confer tissue protection against oxidant injury in other vital organs such as liver and kidney of rats. In this study, the effects of BSS pretreatment on rat models of carbon tetrachloride (CCl_4_) hepatotoxicity and gentamicin nephrotoxicity were investigated. The findings showed that BSS pretreatment protected against CCl_4_-induced hepatotoxicity, but not gentamicin nephrotoxicity in rats. The hepatoprotection afforded by BSS was associated with the improvement in mitochondrial glutathione redox status, presumably through the glutathione reductase-mediated enhancement in mitochondrial glutathione redox cycling. The hepatoprotection afforded by BSS was also accompanied by the improved mitochondrial functional ability in rat livers. The inability of BSS to protect against gentamicin nephrotoxicity was likely due to the relatively low bioavailability of BSS in rat kidneys. BSS may serve as potential mitohormetic agent for the prevention of oxidative stress-induced injury in livers.

## 1. Introduction

Kidney and liver are vital organs that participate actively in metabolic homeostasis, during which a number of reactive oxygen species (ROS) generating reactions are involved. The metabolic role of these organs makes them more vulnerable to oxidant injury [1,2,3,4]. In this connection, experimental findings showed that events such as ethanol consumption [5], drug exposure [6], acute exercise and aging [7,8] were found to be associated with a shift of cellular redox environment to a more oxidized state in the kidney and liver, indicative of oxidative stress, with resultant impairment in cellular and mitochondrial functions [9]. Oxidative stress has been implicated in the pathogenesis of diseases such as chronic renal failure and hepatic fibrosis as well as the aging process [10,11,12,13]. As a consequence, interventions aimed at enhancing cellular antioxidant capacity, preferably by up-regulating cellular antioxidant defense mechanism [14], represent a rational approach for preventing the oxidative stress-induced tissue damage and retarding the aging process.

Cistanches Herba, the dried whole plant of *Cistanche deserticola* Y.C. Ma, is a “Yang-invigorating” tonic herb in Chinese medicine. Recently, a phytosterol, β-sitosterol (BSS, Figure S1, Table S1), was identified as an active component of Cistanches Herba in enhancing mitochondrial respiration and thereby improving glutathione redox status via an induction of mitochondrial ROS production in H9c2 cardiomyocytes. Our findings also demonstrated that BSS protected against oxidant injury in H9c2 cardiomyocytes [15,16]. Therefore, it was postulated that BSS increased mitochondrial electron transport and the associated sustained low level of mitochondrial ROS production in H9c2 cardiomyocytes. The increased mitochondrial ROS production was paralleled by an induction of mitochondrial uncoupling. The elevated level of mitochondrial ROS also caused an up-regulation of cellular glutathione redox cycling and the subsequent increased resistance to oxidant challenge [16]. The beneficial effects of BSS were further corroborated by their ability to protect against myocardial ischemia/reperfusion (I/R) injury *ex vivo* in rats, presumably via enhancing mitochondrial glutathione redox cycling [15,16,17]. Here, we endeavored to investigate whether BSS can also produce tissue protection against oxidant injury in rat livers and kidneys.

Carbon tetrachloride (CCl_4_) hepatotoxicity and gentamicin nephrotoxicity are well established animal models to investigate potential hepatoprotective and nephroprotective agents [18,19,20,21,22,23]. In the present study, the effects of BSS pretreatment on CCl_4_ hepatotoxicity and gentamicin nephrotoxicity were investigated in rats. Mitochondrial glutathione redox status and mitochondrial functional ability in rat kidney and liver tissues were also examined.

## 2. Results

### 2.1. Effects of BSS on CCl_4_ Hepatotoxicity in Rats

**Figure 1:**
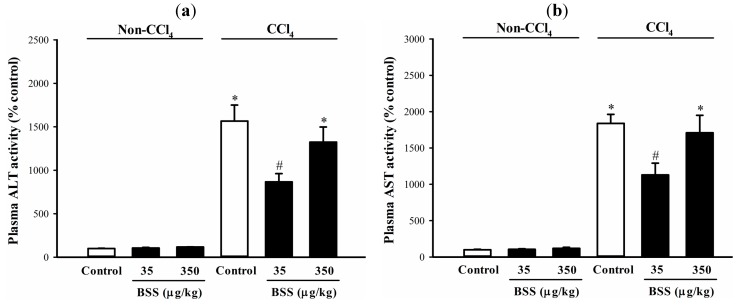
Effects of BSS on plasma ALT and AST activities in control and CCl_4_-intoxicated female rats. Plasma (**a**) ALT and (**b**) AST activity were measured as described in Materials and methods. Data were expressed in percent control with respect to the non-CCl_4_ control (plasma ALT activity = 41.2 ± 7.6 mU/L; plasma AST activity = 73.3 ± 11.8 mU/L). Values given are means ± SEM, with n = 7.

**Figure 2:**
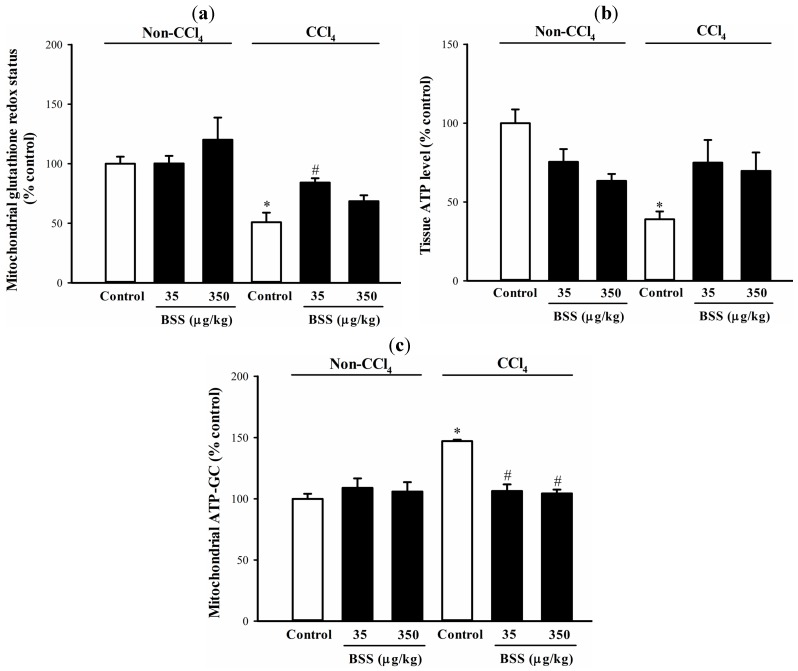
Effects of BSS on control and CCl_4_-intoxicated female rat livers. Data were expressed in percent control with respect to the non-CCl_4_ control ((**a**) mitochondrial GSH/GSSG ratio = 9.6 ± 0.3; (**b**) tissue ATP level = 10.2 ± 0.3 nmol/mg protein; (**c**) ATP-GC value = 999.8 ± 65.7 AU). Values given are means ± SEM, with n = 7.

CCl_4_ administration caused liver injury in rats, as indicated by significant increases in plasma alanine aminotransferase (ALT) and aspartate aminotransferase (AST) activities (by 14 and 16-fold, respectively), when compared with the non-CCl_4_ control (Figure 1). BSS (35 µg/kg) pretreatments protected against CCl_4_ hepatotoxicity, as evidenced by significant suppressions in the CCl_4_-induced elevation in plasma ALT and AST activities by 48% and 41%, respectively, when compared with the respective untreated CCl_4_ control (Figure 1). CCl_4_-induced liver damaged was associated with a significant impairment in the mitochondrial glutathione redox status (by 49%), as assessed by the reduced glutathione (GSH)/oxidized glutathione (GSSG) ratio, in rat livers (Figure 2a). While BSS pretreatment produced no detectable effect on mitochondrial glutathione redox status in non-CCl_4_-intoxicated rat livers (Figure 2a), BSS, at daily doses of 35 µg/kg, partially reversed the CCl_4_-induced impairment in mitochondrial glutathione redox status by 61%, when compared with the untreated CCl_4_ control (Figure 2a).

* Significantly different from the non-CCl_4_ control; ^#^ significantly different from the CCl_4_ control (*p* < 0.05).

Both BSS and CCl_4_ challenge did not alter mitochondrial glutathione reductase (GR) and isocitrate dehydrogenase (ICDH) activities (Table 1). BSS (35 µg/kg) pretreatment increased the mitochondrial ICDH activity by 18% in CCl_4_-intoxicated rat livers, when compared with the unpretreated CCl_4_ control (Table 1).

* Significantly different from the non-CCl_4_ control; ^#^ significantly different from the CCl_4_ control (*p* < 0.05).

**Table 1:** Effects of BSS pretreatment on mitochondrial GR and ICDH activity in livers of CCl_4_-intoxicated rats.

% Non-CCl_4_ Control	Non-CCl_4_ Control	CCl_4_		
		Control	BSS (35 µg/kg)	BSS (350 µg/kg)
Mitochondrial GR Activity	100.0 ± 2.9	98.2 ± 5.1	92.9 ± 9.5	82.8 ± 9.2
Mitochondrial ICDH Activity	100.0 ± 2.2	104.5 ± 3.4	117.5 ± 6.0 *^,#^	102.1 ± 3.7

Data were expressed in percent control with respect to the non-CCl_4_ control (mitochondrial GR activity = 7.1 ± 0.3 mU/mg protein; mitochondrial ICDH activity = 49.8 ± 1.2 mU/mg protein). Values given are means ± SEM, with n = 7. * Significantly different from the non-CCl_4_ control; ^#^ significantly different from the CCl_4_ control (*p* < 0.05).

BSS (350 µg/kg) pretreatment significantly decreased the tissue ATP level by 37% in rat livers under non-challenged condition (Figure 2b). CCl_4_ intoxication caused significant reductions in tissue ATP level by 68% in rat livers (Figure 2b). BSS (35 and 350 µg/kg) pretreatment partially reversed the CCl_4_-induced depletion of tissue ATP by 60 and 51%, respectively, in rat livers (Figure 2b and Table 2). 

BSS treatment produced no detectable change in mitochondrial ATP generation capacity (ATP-GC) in livers of non-CCl_4_-intoxicated rats. Mitochondrial ATP-GC was increased after the CCl_4_ challenge by 55%, when compared with the non-CCl_4_ control (Figure 2c). The CCl_4_-induced elevation in mitochondrial ATP-GC was completely suppressed by BSS pretreatment (Figure 2c).

**Table 2:** Effects of BSS pretreatment on CCl_4_-induced hepatic ATP depletion in rats.

%	CCl_4_		
	Control	BSS (35 µg/kg)	BSS (350 µg/kg)
**CCl_4_-induced Hepatic ATP Depletion**	68.0 ± 4.9	0.5 ^#^ ± 15.4	−6.4 ^#^ ± 12.5

Data were expressed in percent control with respect to respective non-CCl_4_ animals. Values given are means ± SEM, with n = 7. ^#^ Significantly different from the CCl_4_ control (*p* < 0.05).

### 2.2. Effects of BSS on Gentamicin Nephrotoxicity in Rats

**Figure 3:**
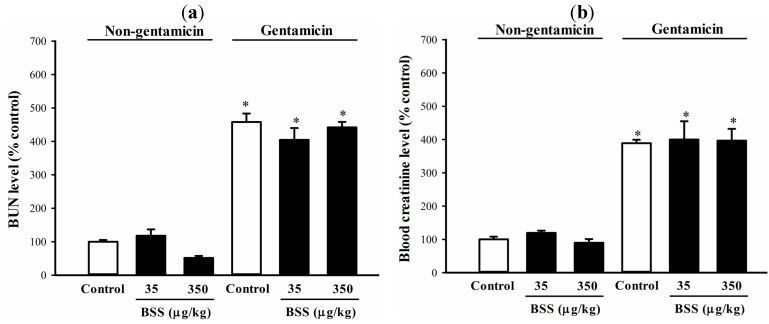
Effects of BSS on BUN and blood creatinine level in control and gentamicin-challenged rats. (**a**) BUN and (**b**) blood creatinine level were measured as described in Materials and methods. Data were expressed in percent control with respect to the non-gentamicin control (BUN level = 187.2 ± 16.2 mg/L; blood creatinine level = 4.5 ± 0.3 mg/L). Values given are means ± SEM, with n = 7.

Gentamicin administration caused kidney injury in rats, as indicated by significant increases in blood urea nitrogen (BUN) and blood creatinine levels (by 3 and 2-fold, respectively), when compared with the non-gentamicin control (Figure 3). BSS produced no detectable effect on the gentamicin-induced elevations in BUN and blood creatinine level (Figure 3).

* Significantly different from the non-gentamicin control (*p* < 0.05).

**Figure 4:**
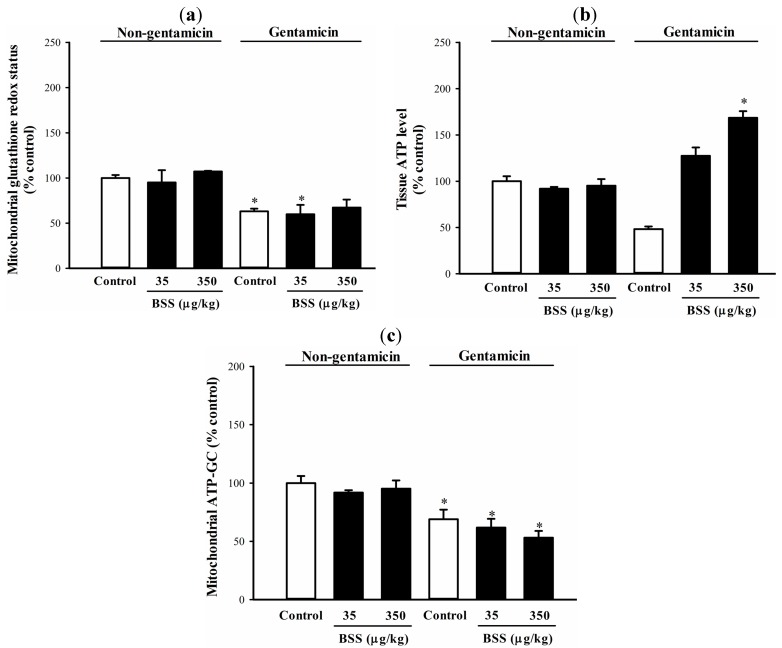
Effects of BSS on control and gentamicin-challenged rat kidneys. (**a**) Mitochondrial glutathione redox status, (**b**) tissue ATP level and (**c**) mitochondrial ATP-GC were examined as described in Materials and methods. Data were expressed in percent control with respect to non-gentamicin control (mitochondrial GSH/GSSG ratio = 13.9 ± 1.6; tissue ATP level = 0.9 ± 0.1 nmol/mg protein; ATP-GC value = 997.8 ± 14.4 AU). Values given are means ± SEM, with n = 7.

The gentamicin-induced kidney injury was found to be associated with a significant impairment in mitochondrial glutathione redox status, as indicated by a decrease (38%) in the ratio of GSH to GSSG (Figure 4a). Consistent with the absence of nephroprotective effect, BSS pretreatment produced no detectable effect on mitochondrial glutathione redox status in rat kidneys under both non-challenged and challenged conditions, when compared with respective unpretreated controls (Figure 4a). Gentamicin challenge also significantly inhibited mitochondrial GR (40%) and ICDH (45%) activities in rat kidneys (Table 3). BSS pretreatment failed to produce any detectable effect on the gentamicin-induced suppression in mitochondrial GR and ICDH activity (Table 3).

* Significantly different from the non-gentamicin control; ^#^ significantly different from the gentamicin control (*p* < 0.05).

**Table 3:** Effects of BSS pretreatment on mitochondrial GR and ICDH activity in kidneys of gentamicin-challenged rats.

% Non-gentamicin Control	Non-gentamicin Control	Gentamicin		
		Control	BSS (35 µg/kg)	BSS (350 µg/kg)
Mitochondrial GR Activity	100.0 ± 4.0	59.6 ± 6.1 *	63.2 ± 3.9 *	61.7 ± 4.7 *
Mitochondrial ICDH Activity	100.0 ± 3.6	54.5 ± 8.0 *	61.0 ± 6.5 *	47.5 ± 2.8 *

Data were expressed in percent control with respect to the non-gentamicin control (mitochondrial GR activity = 11.6 ± 0.5 mU/mg protein; mitochondrial ICDH activity = 17.9 ± 0.5 mU/mg protein). Values given are means ± SEM, with n = 7. * Significantly different from the non-gentamicin-challenged control (*p* < 0.05).

Gentamicin challenge caused a significant depletion of tissue ATP level (by 57%) in rat kidneys, when compared with the respective non-gentamicin control (Figure 4b). While BSS pretreatments produced no detectable change in tissue ATP level in rat kidneys under non-challenged condition (Figure 4b), it (35 and 350 µg/kg) caused increases in tissue ATP level (by 30% and 68%, respectively) over the non-gentamicin control value in gentamicin-challenged rat kidneys (Figure 4b and Table 4). In regard to mitochondrial function, gentamicin challenge caused a significant decrease in mitochondrial ATP-GC by 35% in rat kidneys, when compared with the respective unpretreated non-gentamicin control (Figure 4c). BSS pretreatment did not produce detectable change in mitochondrial ATP-GC in rat kidneys under both non-challenged and challenged conditions, when compared with respective unpretreated controls (Figure 4c).

**Table 4:** Effects of BSS pretreatment on gentamicin-induced renal ATP depletion in rats.

%	Gentamicin		
	Control	BSS (35 µg/kg)	BSS (350 µg/kg)
Gentamicin-induced Renal ATP Depletion	57.1 ± 2.6	−35.6 ^#^ ± 8.4	−73.4 ^#^ ± 7.1

Data were expressed in percent control with respect to respective non-gentamicin-challenged animals. Values given are means ± SEM, with n = 7. ^#^ Significantly different from the gentamicin-challenged control (*p* < 0.05).

## 3. Discussion

BSS was previously identified as an active component of Cistanches Herba in enhancing mitochondrial respiration and thereby inducing mitochondrial glutathione redox cycling, presumably via the intermediacy of mitochondrial ROS production in H9c2 cardiomyocytes and rat hearts [15,16] (Figure S1, Table S1). Previous findings have also demonstrated that long-term treatment with BSS produced protective effect against myocardial I/R injury in rats [15,16]. However, whether BSS pretreatment can protect against oxidant injury in other organs remains unclear. In the present study, we investigated the potential beneficial effects of BSS on oxidant injury in livers and kidneys of rats. The results showed that pretreatments with BSS protected against CCl_4_ hepatotoxicity but not gentamicin nephrotoxicity in rats. The hepatoprotection against CCl_4_ challenge in rat livers was associated with the enhancement in mitochondrial glutathione redox status. It has been demonstrated that a pro-oxidant shift in mitochondrial glutathione redox status (*i.e.*, GSH/GSSG ratio) is associated with age-related cell death [24] and oxidative stress-induced tissue damages [25,26,27,28], presumably due to a disruption of redox signaling cascades. The maintenance of an optimal glutathione redox status is therefore critical for cell survival [29]. This postulation is consistent with our finding that the degree of tissue protection correlated with the extent of enhancement of mitochondrial glutathione redox status in BSS-pretreated CCl_4_-intoxicated rat livers. The hepatoprotective effect of BSS may be mediated by an up-regulation of mitochondrial glutathione redox cycling, which involves the regeneration of GSH from GSSG. In cellular environment, GSH is readily oxidized to GSSG for ROS detoxification, and it can be regenerated via a GR-catalyzed reaction at the expense of NADPH. In this regard, BSS pretreatment was found to increase mitochondrial ICDH activity in CCl_4_-intoxicated rat livers, wherein mitochondrial ICDH plays a pivotal role in supplying NADPH for the regeneration of GSH from GSSG and the subsequent improvement in mitochondrial glutathione redox status via the action of mitochondrial GR [30,31,32]. The degree of stimulation of ICDH activity was also found to correlate with an improvement in mitochondrial glutathione redox status in BSS-pretreated challenged animals, suggesting that BSS-induced up-regulation of mitochondrial glutathione redox cycling may be mediated by the activation of mitochondrial ICDH activity in rat livers. On the other hand, gentamicin-induced oxidant injury was found to be associated with the suppressions of mitochondrial GR and ICDH activities in rat kidneys. The absence of such association in CCl_4_-intoxicated rat livers may be explained by the differential susceptibility of kidney and liver to oxidant challenges [33,34], as was also observed in the present study. The incapability of BSS to ameliorate the gentamicin-induced impairment in mitochondrial glutathione redox status and suppression in mitochondrial GR and ICDH activities further supported the importance role of mitochondrial glutathione redox status in tissue protection against oxidant injury.

The observation that BSS induced hepatic ATP depletion under non-challenged condition indicated a sustained induction of mitochondrial uncoupling, whereas BSS failed to produce any effect on tissue ATP level in rat kidneys. The discrepant observation between livers and kidneys in regard to the effect of BSS on tissue ATP in non-challenged rats may be attributed at least in part to a relatively high resistance of kidney tissue to ATP depletion, as was the case in metabolic insults [35]. Both CCl_4_ and gentamicin challenge caused significant depletions in tissue ATP levels in rat livers and kidneys, presumably due to an impaired mitochondrial electron transport and oxidative phosphorylation. While BSS produced no detectable effect on mitochondrial ATP-GC in rat livers, the hepatoprotective effect of BSS was found to be associated with the reduction in the extent of CCl_4_-induced tissue ATP depletion (Table 2), which may be explained by a reduced extent of tissue/mitochondrial damage under oxidative stress conditions. The unexpected increases in renal ATP level in BSS-pretreated and gentamicin-challenged rats remain to be investigated (Figure 4b and Table 4). It is possible, as reported by Devalaraja-Narashimha *et al.* [36] and Park *et al.* [37], that BSS may modulate renal energy homeostasis by its ability to degrade poly-(ADP-ribose) polymerase, an enzyme that induces a significant ATP depletion and cell death under oxidative stress condition.

Our previous findings on H9c2 cardiomyocytes showed that the cytoprotective effect afforded by HCF1 (an active fraction of Cistanches Herba)/BSS against hypoxia/reoxygenation-induced apoptosis was mediated by the induction of mitochondrial glutathione redox cycling via the HCF1-induced mitochondrial ROS production secondary to increased mitochondrial electron transport [15,16]. Based on our current findings, we postulate that BSS pretreatment may up-regulate mitochondrial glutathione redox cycling by increasing mitochondrial ROS production, possibly through the stimulation of mitochondrial electron transport. Preliminary studies indicated that BSS may increase the fluidity of mitochondrial inner membrane and thereby stimulate mitochondrial electron transport (data not shown). The finding that BSS protected against CCl_4_ hepatotoxicity suggests its potential use as a mitohormetic agent to prevent oxidative stress-induced tissue injury in the liver, particularly during the aging process, in which increased prevalences of various liver diseases were observed [38,39,40]. While the BSS-containing HCF1 could protect against gentamicin nephrotoxicity in rats (unpublished data), the inability of BSS in ameliorating the gentamicin nephrotoxicity may be explained by its relatively low bioavailability, particularly when in the absence of other components in HCF1, in rat kidneys. It has been shown that BSS as well as other phytosterols, such as campesterol, exhibited a relatively low concentration and a short retention time in rat kidneys, when compared with rat livers [41]. While dietary compounds such as polyphenols and polyunsaturated fatty acids have been shown to improve mitochondrial function and regulate apoptosis process both in normal and pathological conditions [17], our recent and present findings of enhancement of mitochondrial ATP generation capacity and glutathione redox status by BSS demonstrate the potential beneficial effect of triterpenoids on mitochondrial energy generation and antioxidant mechanism [16].

## 4. Experimental Section

### 4.1. Chemicals

BSS (CAS 83-46-5) was purchased from Sigma (St. Louis, MO, USA). QuantiChrom^TM^ Urea Assay kit (DIUR-500) and QuantiChrom^TM^ Creatinine Assay kit (DICT-500) were purchased from BioAssay Systems (Hayward, CA, USA). ATPLite^TM^ bioluminescence assay kit (#6016941) was purchased from Perkin Elmer Inc. (Santa Clara, CA, USA). Assay kits for ALT activity and AST activity, and all other chemicals were purchased from Sigma.

### 4.2. Animal Care

Female Sprague-Dawley rats (8 weeks; 200 to 250 g) were maintained under a 12-h dark/light cycle in an air/humidity controlled room at about 22 °C, and allowed food and water *ad libitum* in the Animal and Plant Care Facilities (APCF) at HKUST. All experimental procedures were approved by the Research Practice Committee (HKUST) [42]. Rats were randomly divided into groups, with seven animals in each unless otherwise specified. In the experiment, rats were administered intragastrically with BSS, at daily doses of 35 and 350 µg/kg, which were proven to be effective to protect against myocardial ischemia/reperfusion injury in rats [16], for 14 consecutive days. Control animals received vehicle (olive oil) only.

### 4.3. CCl_4_ Hepatotoxicity

CCl_4_ was intragastrically administered at a dose of 1 mL/kg after the last dosing of BSS, and vehicle was given to the non-CCl_4_-intoxicated rats. Twenty-four hours after CCl_4_ challenge, blood samples were drawn from phenobarbital-anesthetized animals by cardiac puncture and the animals were then sacrificed by cardiac excision. The blood samples were used for measuring plasma ALT and AST using assay kits. Liver tissue samples were obtained and subjected to biochemical analysis.

### 4.4. Gentamicin Nephrotoxicity

Gentamicin was administered intraperitoneally at a daily dose of 150 mg/kg from day 9 to day 14 during the course of BSS treatment (6 doses). Vehicle was given to the non-gentamicin-challenged animals. Twenty-four hours after the last gentamicin injection, blood samples were drawn from phenobarbital-anesthetized animals by cardiac puncture. The blood samples were used for measuring BUN and plasma creatinine levels, using assay kits. Animals were then sacrificed by cardiac excision. Both kidneys were harvested and subjected to biochemical analysis.

### 4.5. Tissue ATP Level

Tissue homogenates (10%, w/v) were prepared by homogenizing the minced tissues with a Teflon-glass homogenizer at 4000 rpm for 10 complete strokes. An aliquot (20 µL) of 30% (v/v) perchloric acid (PCA) was added to 100 µL of tissue homogenates. After neutralization by potassium bicarbonate, the ATP content was measured by bioluminescence assay. The data were normalized with respective protein content and expressed as nmol ATP per mg tissue protein.

### 4.6. Mitochondrial Fraction Preparation

Mitochondrial fractions were prepared by differential centrifugation in corresponding isotonic buffers (kidney: 320 mM sucrose, 1 mM ethylenediaminetetraacetic acid (EDTA), 50 mM Tris/HCl, pH 7.4; liver: 250 mM sucrose, 0.1 mM EDTA, 5 mM Tris/HCl, pH7.4), as previously described [43].

### 4.7. Mitochondrial ATP-GC ex Vivo

Mitochondrial fraction (adjusted to 1 mg protein/mL) was mixed with substrate solution (3 mM pyruvate and 3 mM malate) and ADP (30 mM) solution to allow mitochondrial ATP generation. The ATP content was then measured as described [44].

### 4.8. Mitochondrial Glutathione Redox Status

Mitochondrial GSH and GSSG levels were measured enzymatically using 5,5′-dithiobis-(2-nitrobenzonic acid) (DTNB) and GR as described [45]. The glutathione redox status was assessed by the ratio of GSH/GSSG.

### 4.9. Mitochondrial GR and ICDH Activities

Mitochondrial GR activity was measured as described by Chiu *et al.* (2003) [46]. For the measurement of mitochondrial ICDH activity, a sample mixture, which was prepared by mixing mitochondrial fraction (100 µL), 0.3% Triton-X (100 µL) and phosphate-buffered saline (100 µL), was sonicated at 4 °C for 10 min. An aliquot (120 µL) of reaction mixture (5 mM isocitrate, 0.435 mM NADP and 1 drop of 0.1 M MnCl_2_) was added to the sample mixture (60 µL). Absorbance changes at 340 nm were monitored at 37 °C for 5 min. ICDH activity was estimated by noting the absorption coefficient of NADPH.

### 4.10. Statistical Analysis

All data were expressed as mean ± standard error of the mean (SEM), unless otherwise specified. Data were analyzed by one-way analysis of variance (one-way ANOVA) and inter-group difference was detected by Turkey range test when *p* < 0.05.

## 5. Conclusions

In conclusion, BSS pretreatment protected against CCl_4_ hepatotoxicity but not gentamicin nephrotoxicity in rats. The hepatoprotection afforded by BSS against oxidant injury is likely mediated by the up-regulation of mitochondrial glutathione redox cycling via the induction of mitochondrial ROS production in rat livers. Therefore, long-term treatment with BSS may produce a mitohormetic action to prevent oxidative stress-induced oxidant injury in the liver.

## Abbreviations

ALT: Alanine aminotransferase
AST: aspartate aminotransferase
ATP-GC: ATP generation capacity
BSS: β-sitosterol
BUN: blood urea nitrogen
CCl_4_: carbon tetrachloride
DTNB: 5,5'-dithiobis-(2-nitrobenzoic acid)
EDTA: ethylenediaminetetraacetic acid
GR: glutathione reductase
HCF1: Cistanches Herba fraction
ICDH: isocitrate dehydrogenase
GSSG: oxidized glutathione
PCA: perchloric acid
ROS: reactive oxygen species
GSH: reduced glutathione

## Supplementary Materials

Supplementary materials can be accessed at: http://www.mdpi.com/1420-3049/19/11/17649/s1.

## Supplementary Files

Supplementary File 1
