# Supplementary material for: β-Sitosterol Protects against Carbon Tetrachloride Hepatotoxicity but not Gentamicin Nephrotoxicity in Rats via the Induction of Mitochondrial Glutathione Redox Cycling
Source: Molecules. 2014 Oct 30;19(11):17649–62. doi: 10.3390/molecules191117649 (PMC6271253; doi:10.3390/molecules191117649)

Supplementary Materials

Table S1. Chemical characterization of phytosterols in HCF1 by HPLC-MS/MS.

Peak no.	Retention Time (min)	$[M+H]^+$	$[M+Na]^+$	$[M+K]^+$	MS/MS	Identification	Quantitative Estimation (% Area)
1	29.77	401	423	439	383 $[M+H-H_2O]^+$; 161	CAMP	13.9
2	32.94	415	-	-	397 $[M+H-H_2O]^+$; 161	BSS	2.7
3	33.97	417	439	455	399 $[M+H-H_2O]^+$; 163	SS	11.6

The quantitative estimation of CAMP, BSS and SS were expressed in percent area with respect to the total chemical content of HCF1.

Figure S1. TIC chromatography of Herba Cistanches Fraction One (HCF1), a semi-purified fraction of Cistanches Herba by HPLC-MS/MS. The TIC chromatography of HCF1 was obtained as described in Materials and methods. Data were expressed as relative abundance of particular chemical constituents (1: campesterol (CAMP); 2: BSS and 3: sitostanol (SS)).

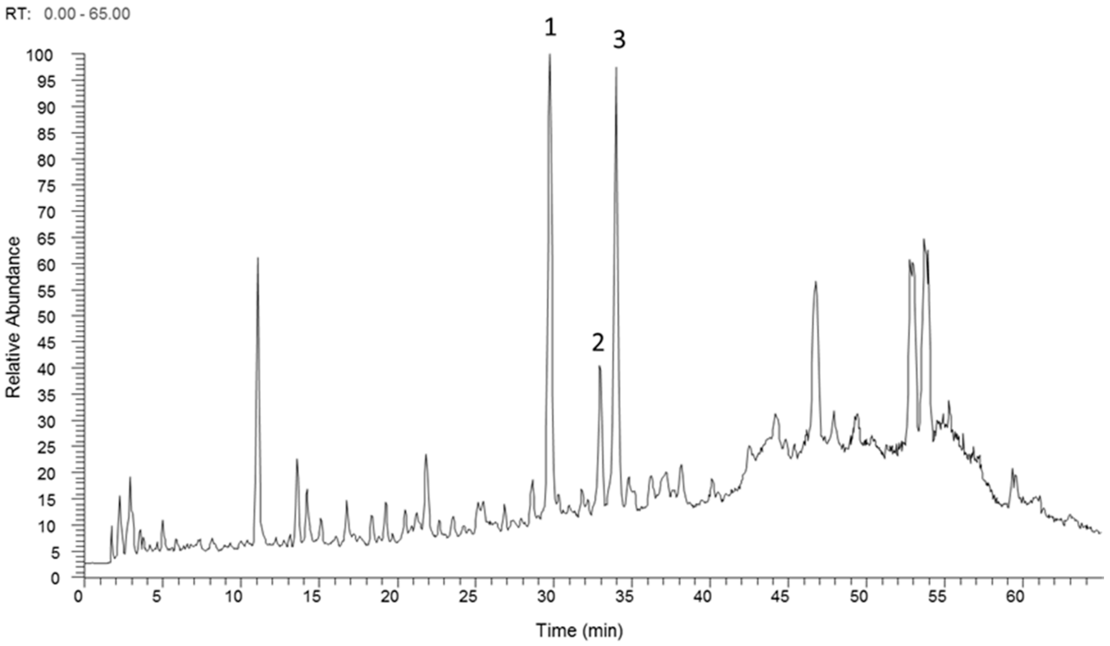

Supplement: Supplementary File 1 [file molecules-19-17649-s001.pdf]
